# Enhancing Prognostic Signatures in Glioblastoma with Feature Selection and Regularised Cox Regression

**DOI:** 10.3390/genes16050473

**Published:** 2025-04-23

**Authors:** Beatriz N. Leitão, André Veríssimo, Alexandra M. Carvalho, Susana Vinga

**Affiliations:** 1 Instituto de Engenharia de Sistemas e Computadores: Investigação e Desenvolvimento (INESC-ID), Instituto Superior Técnico, Universidade de Lisboa, 1000-029 Lisbon, Portugal; 2 Instituto de Telecomunicações (IT–Lisboa), Instituto Superior Técnico, Universidade de Lisboa, 1049-001 Lisbon, Portugal; 3 Appsilon, Data for Good, 00-020 Warsaw, Poland; 4 Instituto de Engenharia Mecânica (IDMEC), Instituto Superior Técnico, Universidade de Lisboa, 1049-001 Lisbon, Portugal

**Keywords:** survival analysis, prognostic models, machine learning, network-based regularisation, precision oncology, cancer biomarkers, TCGA

## Abstract

Background: Glioblastoma is a highly aggressive brain tumour with poor survival outcomes, highlighting the need for reliable prognostic models. Developing robust and interpretable prognostic signatures is critical for improving patient stratification and guiding therapy. This study explored the integration of machine learning feature selection with regularised Cox regression to construct prognostic gene signatures for glioblastoma patients. Methods: We combined the Boruta algorithm and Random Survival Forests (RSFs) with regularised Cox regression, along with network-based regularisation techniques (HubCox and OrphanCox), to develop interpretable prognostic signatures for stratifying high- and low-risk glioblastoma patients. Using mRNA-seq and survival data from The Cancer Genome Atlas (TCGA), we developed predictive models following WHO-2021 glioma guidelines. Results: Integrating Boruta or RSF with regularised Cox regression improved the performance and interpretability. Boruta increased the concordance indexes (C-indexes) by 0.030 and 0.013 for LASSO and Elastic Net, respectively, while significantly reducing the feature numbers. RSF similarly enhanced the performance and feature reduction. The genes Lysyl Oxidase Like 1 (*LOXL1*) and Insulin Like Growth Factor Binding Protein 6 (*IGFBP6*) were consistently selected and linked to glioma survival, emphasising their clinical significance. The network-based methods demonstrated superior survival probability prediction (lower Integrated Brier Score), although with lower C-index values, highlighting limitations in ranking the survival times. To evaluate the generalisability, external validation using the Chinese Glioma Genome Atlas (CGGA) confirmed that a multigene signature derived from the most consistently selected genes significantly stratified the patients by risk. Conclusions: This study underscored the utility of combining machine learning feature selection with survival analysis to enhance prognostic modelling while balancing predictive performance and interpretability.

## 1. Introduction

Brain tumours are associated with high morbidity and mortality rates. Gliomas, which are thought to arise from neuroglial stem or progenitor cells, account for nearly 30% of all primary brain tumours and 80% of malignant ones, causing the majority of deaths related to primary brain cancers [1].

In recent years, significant progress has been made in understanding the molecular pathogenesis of gliomas. Identifying tumour groups exhibiting varying clinical presentations and survival patterns has led to major updates to the tumour classification system in the 2016 and 2021 editions of the World Health Organization Classification of Tumours of the Central Nervous System (WHO-2016 and WHO-2021). The WHO-2016 guidelines incorporated genetic and molecular information alongside traditional histological evaluation, refining the diagnosis of gliomas. This trend continued with the WHO-2021 guidelines, which placed even greater emphasis on genetic markers and molecular profiles for determining diagnosis and prognosis, moving away from the traditional histogenetic classification [2,3].

Molecular studies of gliomas have mainly been supported by open-access databases, such as The Cancer Genome Atlas (TCGA) database [4]. TCGA provides extensive data on various cancer types, including WHO grade II and III lower-grade gliomas (LGGs) and grade IV glioblastomas (GBMs). However, the TCGA datasets, collected until 2013, assigned glioma cases to histological subtypes, like mixed oligoastrocytic gliomas, which are now discouraged in clinical practice. As a result, these classifications do not consistently align with the WHO-2016 and WHO-2021 taxonomy guidelines. New methodologies have been proposed to address the challenges of outdated diagnostic annotations in adult glioma samples. These methodologies aim to update tumour classifications effectively into astrocytoma and oligodendroglioma (both LGGs) and GBM. This ensures alignment with current diagnostic standards and enhances the clinical applicability [2].

Even with advancements in therapy, glioma patients face poor survival rates. The median overall survival for GBM is 9.2 months [5], whereas LGG has a median overall survival of 7 years [6]. This highlights the importance of identifying new and effective signatures to improve the diagnosis and outcome prediction for these patients, as it can potentially improve patient management and counselling. For instance, the recently proposed DAK-75 scoring system [7] introduces a prognostic score to guide surgical decision-making in elderly patients with high-grade gliomas, exemplifying the clinical utility of such tools. These approaches demonstrate the potential of data-driven methods to personalise treatment strategies and support decision-making in complex clinical scenarios.

In recent years, numerous studies have explored the application of the Cox proportional hazards regression model [8], commonly known as Cox regression, and regularisation techniques to develop prognostic signatures for GBM based on mRNA expression data [9,10,11,12]. Many of these studies tended to follow a similar workflow, which often included screening survival-associated genes using univariate Cox regression, followed by refinement with regularised multivariate Cox regression. Univariate and multivariate Cox regressions have long been cornerstone techniques for identifying gene signatures that predict GBM prognosis. These methods are often enhanced with regularisation techniques, such as the Least Absolute Shrinkage and Selection Operator (LASSO) [13]. Regularisation may reduce overfitting and enhance the model interpretability by shrinking the coefficients of less relevant features. The prognostic signatures obtained using these models are often constructed using a few genes and their corresponding coefficients, providing a clear understanding of how each gene contributes to patient outcomes. This clarity facilitates the integration of biological insight into clinical decision-making.

For instance, Liu et al. [9] developed a six-mRNA signature to predict survival in GBM patients by analysing genome-wide mRNA profiles. They initially used univariate Cox regression to filter survival-associated genes, followed by LASSO-regularised multivariate Cox regression (LASSO–Cox) to construct a refined risk score model.

Gong et al. [10] followed a similar workflow but first identified differentially expressed genes (DEGs) between patients with good and poor prognoses. Prognostic mRNAs were then selected using univariate Cox regression, followed by further refinement with LASSO–Cox, which resulted in an eight-mRNA prognostic signature.

Similarly, Prasad et al. [11] identified DEGs in GBM compared with normal brain tissues through a meta-analysis of microarray datasets and RNA-seq data. They selected common DEGs and then screened for prognosis-related genes using univariate Cox regression analysis. Subsequently, they applied LASSO–Cox regression, which resulted in a survival-associated four-gene signature.

Finally, Brandão et al. [12] began with univariate variable selection, followed by multivariate Cox regression using three regularisation techniques, namely, Elastic Net [14] and the methods HubCox and OrphanCox introduced by Veríssimo et al. [15], to refine the variable selection. A univariate Cox model was then built for all variables selected by each regularisation method, with variables that had a Wald test q-value below 0.20 retained for the final Cox survival model. In addition to developing the prognostic signature, their analysis included an outlier detection step using the rank product test [16], which identified patients with consistently large martingale residuals across models, highlighting potential outliers.

These studies demonstrate the power of combining Cox regression and regularisation methods to create concise, interpretable models for survival prediction while leveraging biological knowledge to deepen the understanding of disease mechanisms.

Emerging statistical and machine learning computational approaches, such as Random Survival Forests (RSFs) [17] and the Boruta algorithm [18], provide promising tools for biomarker discovery and hold significant potential for advancing glioma research. However, these approaches are not yet widely adopted due to challenges in interpretability, as models like the RSF often operate as black boxes, making it difficult to understand how individual features contribute to predictions.

In our study, we extended the regularised multivariate Cox regression approach by incorporating RSF [17] and the Boruta algorithm [18] alongside regularised Cox regression. This combination allowed us to leverage the robust predictive performance and advanced feature selection capabilities of these methods while preserving the interpretability of Cox regression models. We applied this methodology to the TCGA dataset, following the WHO-2021 taxonomy guidelines as implemented by Mendonça et al. [2], and further evaluated the generalisability of the resulting gene signature through external validation on the Chinese Glioma Genome Atlas (CGGA) dataset [19]. Our objective was not only to develop an innovative methodology to improve prognostic modelling for GBM but also to identify possible relevant genes that may serve as potential biomarkers or therapeutic targets, which can be further validated in future clinical and experimental studies.

## 2. Materials and Methods

### 2.1. Data Collection and Pre-Processing

In our research, mRNA-seq data and survival data from LGG and GBM patients were obtained from the TCGA database using the TCGAbiolinks package (version 2.30.0) [20]. The inclusion criteria were as follows: (1) patients diagnosed with LGG/GBM, (2) patients who had mRNA-seq data and (3) patients who had survival data. Among them, there were 160 GBM patients and 510 LGG patients. Afterwards, the disease classification of the patients was updated using the WHO-2021 taxonomy guidelines already implemented and made available by Mendonça et al. [2]. Notably, the number of GBM cases increased after applying the new classification. This was because under the updated WHO-2021 classification, some patients who were previously classified as having LGG were now reclassified as having GBM. As a result, the final dataset included 213 GBM patient samples.

Given the highly aggressive nature of GBM and its poor survival outcomes, we focused exclusively on identifying new and effective molecular signatures to improve the diagnosis and outcome prediction for these patients. The GBM mRNA-seq data were normalised using the trimmed mean of M-values (TMM) method implemented in the edgeR package (version 4.0.15) [21], followed by transformation with the voom function [22].

The GBM patients were randomly divided into two groups: a training set that comprised 70% of the samples for model training and a test set that comprised 30% of the samples for model validation. This data split was performed using the rsample package (version 1.2.1) with stratification to ensure the same proportion of censored data in both sets. To assess the robustness of the models and evaluate the variability in feature selection and performance metrics, the entire pipeline was repeated 10 times, each with a different random split of the data while maintaining the 70%/30% proportion. This iterative approach allowed for a more reliable estimation of the model stability and generalizability.

### 2.2. Cox Regression

Considering the usual survival data setup, let $D=\{\left(X_{1},Y_{1},\delta_{1}\right),\ldots ,\left(X_{n},Y_{n},\delta_{n}\right)\}$ represent the dataset, where $X_{i}$ denotes the gene expression profile of the *i*th patient, i=1,…,n, consisting of *p* genes, such that $X_{i}=\left(X_{i1},\ldots ,X_{ip}\right)$. Here, $Y_{i}$ corresponds to the survival time for patient *i*, and $\delta_{i}$ is a binary indicator that specifies whether the event of interest was observed ($\delta_{i}=1$) or censored ($\delta_{i}=0$). The hazard function for a patient given their expression profile is given by

(1) $$h\left(t|X_{i}\right)=h_{0}\left(t\right)\exp\left(\beta_{1}X_{i1}+\beta_{2}X_{i2}+\ldots +\beta_{p}X_{ip}\right)=h_{0}\left(t\right)\exp\left(X_{i}^{⊤}\beta \right).$$

This formulation is the basis of the *Cox proportional hazards model* [8]. This model operates under the proportional hazards assumption, which implies that the hazard ratios between individuals with different feature values remain constant over time. This means that features have a multiplicative effect on the hazard, but this effect does not change as time progresses. The hazard function indicates the risk of the event occurring at time *t* given the individual’s features and the fact that the event has not occurred up to time *t*. The term $h_{0}\left(t\right)$ is the *baseline hazard function* corresponding to the hazard when all feature values are set to zero and depends only on time *t*. Finally, $\exp(\beta_{1}X_{i1}+\beta_{2}X_{i2}+\cdots +\beta_{p}X_{ip})$ represents the effect of the features on the hazard, where $\beta_{1},\beta_{2},\ldots ,\beta_{p}$ are the regression coefficients estimated from the data. Cox regression does not require the baseline hazard function specification, making it a semi-parametric method.

Cox regression is widely applied to quantify the effect of features on survival outcomes and to derive a *prognostic index* (PI) that summarises an individual’s relative risk. The prognostic index is derived from the linear combination of the features weighted by their corresponding regression coefficients. This index for patient *i* is expressed as

(2) $${\mathrm{PI}}_{i}=\beta_{1}X_{i1}+\beta_{2}X_{i2}+\ldots +\beta_{p}X_{ip}.$$

The prognostic index can be interpreted as a measure of how much an individual’s features contribute to their overall risk relative to others in the population. This ability to calculate a prognostic index makes Cox regression highly valuable for developing predictive models that categorise individuals into different risk groups or for identifying key prognostic factors.

### 2.3. Feature Selection with Ensemble-Based Approaches

To efficiently narrow down the set of features and focus on the most relevant, as a first step, univariate Cox regression analysis was applied to pre-select the overall survival (OS)-associated mRNAs. Using the coxph function from the survival package (version 3.7-0) [23], each feature (mRNA) was analysed independently, and those with a *p*-value below 0.05 were retained for further multivariate analysis. This step ensured that only the features with a statistically significant impact on survival were included by refining the selection process for downstream model construction. A multiple-testing correction was not applied, as we adopted a more conservative approach in the feature selection, minimising the risk of excluding potentially relevant prognostic biomarkers.

After pre-selecting features using univariate Cox regression, the RSF (using the randomForestSRC package, version 3.3.1 [17]) and the Boruta algorithm (using the Boruta package, version 36i11 [18]) were employed to determine the most relevant features.

The Random Survival Forest (RSF) algorithm builds upon the random forest methodology, which was specifically designed to accommodate survival analysis and effectively manage censored data [17]. When using RSF, multiple decision trees are built from bootstrapped samples of the dataset, where each tree is grown using a splitting rule that maximises the difference in survival times between the child nodes. This algorithm incorporates ensemble learning, combining the predictions from all trees to estimate survival probabilities or risk scores for individuals [17].

A key aspect of RSF is its ability to rank feature importance using the Variable Importance (VIMP) score [24]. The VIMP score measures the contribution of a feature to the model’s predictive performance by assessing how modifications to its values affect the model’s predictions. Features with higher VIMP scores have a greater impact on the survival prediction. In this study, these scores were later used to optimise the feature selection threshold; this determined which features were retained for the regularised Cox model training.

The Boruta algorithm is an all-relevant feature selection method that works as a wrapper around machine learning models, including RSF, to handle survival data [18]. By leveraging the feature importance scores generated by the underlying model, Boruta takes an additional step by comparing them to shadow features, which are randomised duplicates of the original ones and serve as a relevance baseline. The algorithm iteratively compares the importance of each original feature to the most critical shadow feature, which should only show nonzero importance due to random fluctuations. Based on this comparison, each feature is assigned one of three labels: *confirmed* if it consistently outperforms the shadow features; *rejected* if it fails to do so; or *tentative* if its importance remains inconclusive, which is often due to fluctuating importance scores or convergence limits.

These machine learning models function as a black boxes, making interpreting the factors driving their predictions challenging. In our work, instead of using the models directly, the most important features identified by each method were subsequently fed into a regularised multivariate Cox regression model to enhance the interpretability. In this way, these models served as feature selection tools by extracting relevant groups of features associated with survival.

### 2.4. Regularised Multivariate Cox Regression

After selecting the features, they were incorporated into a regularised multivariate Cox regression model to construct the prognostic indexes. These prognostic indexes were built using the traditional regularisation methods LASSO [13], Elastic Net [14] and Ridge [25] implemented with the glmnet package (version 4.1-8). In addition to classical regularisation methods, we also applied the network-based regularisation methods HubCox and OrphanCox [15] implemented with the glmSparseNet package (version 1.20.1). For the models that applied HubCox and OrphanCox regularisation, neither univariate Cox regression nor the selection of features using the RSF or the Boruta algorithm was applied.

Regularisation methods help control the model complexity. This is achieved by adding a penalisation term to the partial log-likelihood function of the Cox proportional hazards model, which assesses how well the features explain the observed survival data without assuming a specific form for the baseline hazard function. The partial log-likelihood function is given by

(3) $$\log L\left(\beta \right)=l\left(\beta \right)=\sum_{i=1}^{n}\delta_{i}\left\{X_{i}^{⊤}\beta -\log\left[\sum_{j:y_{j}\geq y_{i}}^{n}\exp\left(X_{j}^{⊤}\beta \right)\right]\right\}.$$

The penalisation shrinks the coefficient estimates towards zero, allowing the model to emphasise the most relevant features while minimising the influence of the less important ones.

LASSO (Least Absolute Shrinkage and Selection Operator) applies the $\ell_{1}$-norm penalty, which leads to some coefficients being shrunk to exactly zero, effectively selecting only the most important features. Ridge regression uses the $\ell_{2}$-norm penalty, which shrinks all coefficients but does not eliminate any, making it useful when all features should contribute to the model. Elastic Net combines both LASSO and Ridge regularisation. It balances the feature selection and coefficient shrinkage by applying a mix of the $\ell_{1}$-norm and $\ell_{2}$-norm penalties, allowing for both feature selection and coefficient shrinkage, making it flexible for datasets with correlated features. The formula for Elastic Net regularisation is

(4) $$\min\left(l\left(\beta \right)-\lambda \left(\alpha \sum_{j=1}^{p}\left|\beta_{j}\right|+\left(1-\alpha \right)\sum_{j=1}^{p}\beta_{j}^{2}\right)\right),$$

where l(β) is the partial log-likelihood function, measuring how well the model fits the data. The parameter λ controls the overall strength of the regularisation, with larger values applying more shrinkage. The parameter α balances between LASSO and Ridge, where α=1 corresponds to LASSO and α=0 corresponds to Ridge. In this study, α=0.5 was used for Elastic Net. The term $|\beta_{j}|$ represents the absolute values of the coefficients, contributing to the $\ell_{1}$-norm (LASSO part), while $\beta_{j}^{2}$ represents the squared values of the coefficients, contributing to the $\ell_{2}$-norm (Ridge part).

HubCox and OrphanCox [15] introduce network-based regularisation techniques by incorporating centrality-based penalties derived from network structures. By leveraging prior knowledge that can be directly estimated from the data or from the protein–protein interaction network (PPI), it incorporates information on gene relationships, such as expression correlations or shared protein interactions. It introduces a graph *G* degree constraint that captures the importance of a gene by considering how highly connected it is within a biological network. In these networks, genes are represented as vertices *V*, and the weight of each edge *E* corresponds to the relationship between connected genes given by G=(V,E). In this context, the nodes are *p* features, with p=|V|, and the graph *G* may also be represented by a p×p positively weighted adjacency matrix denoted by $W={\left\{W_{ij}\right\}}_{1\leq i,j\leq p}$, which is used to quantify the relationships. The degree of each node, $d_{i}$, is defined as

(5) $$d_{i}=\sum_{j=1;j\neq i}^{p}W_{ij}.$$

The penalty function for the network-based regularisation is defined as

(6) $$\min\left(l\left(\beta \right)-\lambda \left(\alpha \sum_{j=1}^{p}\left|w_{j}\beta_{j}\right|+\left(1-\alpha \right)\sum_{j=1}^{p}{\left(w_{j}\beta_{j}\right)}^{2}\right)\right),$$

where $w_{j}$ represents the degree centrality of feature *j* in the network, which is a scaled and transformed version of $d_{j}$, to raise the difference between the nodes with small degrees and those with high degrees. The vector of centrality weights *w* is derived from the graph G=(V,E), which encodes the relationships between features (e.g., genes) based on the correlation or protein–protein interactions [15].

By including the centrality weights $w_{j}$, this formulation adapts the Elastic Net penalty to incorporate the network structure. In HubCox, higher weights are assigned to low-degree nodes, discouraging their selection and focusing the model on hubs. Conversely, in OrphanCox, high-degree nodes are penalised more heavily, enabling the inclusion of orphans, or less-connected nodes, in the model. This dual approach ensures that both highly connected and peripheral features can be analysed depending on the biological objective.

To assess the impact of using RSF, Boruta and network-based regularisers for feature selection, we also applied regularised multivariate Cox regression directly after performing the univariate Cox regression.

### 2.5. Model Evaluation

#### 2.5.1. C-Index

The constructed predictors were evaluated using Harrell’s *concordance index* (C-index) [26]. The C-index is a statistical measure used to evaluate the predictive accuracy of survival models by assessing the model’s ability to predict higher relative risks to individuals whose event occurs first. It checks whether the model can correctly predict who has a higher risk for all possible pairs of individuals. A C-index of 1 means perfect concordance between the predicted and observed survival outcomes, while 0.5 indicates random guessing.

In a survival analysis, certain pairs of observations can not be directly compared. For instance, if one individual is censored at time 5 and another passes away at time 6, we can not tell if the first would have survived beyond the second. As a result, the pool of pairs we can evaluate is reduced. For a pair to be comparable, either both individuals must have experienced the event (death), or one individual must have experienced the event while the other was lost to follow-up after the event occurred in the first. The survival package in R was used to calculate the C-index.

#### 2.5.2. Integrated Brier Score

The Brier Score (BS) [27] quantifies the accuracy of probabilistic predictions by measuring the average difference between observed outcomes and predicted probabilities. Without censored data, it is calculated as the squared difference between a patient’s observed status and the predicted probability. However, in survival analysis, censoring must be accounted for. To handle censoring, Graf et al. [28] introduced a method using the Inverse Probability of Censoring Weights (IPCWs) [29], which requires estimating the censoring survival function, denoted as G(t). For each individual, we observe ${\tilde{T}}_{i}=\min\left(T_{i},C_{i}\right)$, where $T_{i}$ is the time until the event of interest, and $C_{i}$ is the time under observation (i=1,2,…,n). Additionally, $\delta_{i}=I\left(T_{i}\leq C_{i}\right)$ indicates whether the event was observed ($\delta_{i}=1$) or censored ($\delta_{i}=0$). It is assumed that *T* and the covariate *X* are independent of *C*. The distribution of *C* is defined as G(t)=P(C≥t). $S(t^{*}|X_{i})$ is the predicted survival probability at $t^{*}$ for patient *i*. The BS for censored data at time $t^{*}$ is calculated using

(7) $$\mathrm{BS}\left(t^{*}\right)=\frac{1}{n}\sum_{i=1}^{n}\left[\frac{{\left(0-S(t^{*}|X_{i})\right)}^{2}}{G({\tilde{T}}_{i})}\cdot I\left({\tilde{T}}_{i}<t^{*},\delta_{i}=1\right)+\frac{{\left(1-S(t^{*}|X_{i})\right)}^{2}}{G(t^{*})}\cdot I\left({\tilde{T}}_{i}\geq t^{*}\right)\right].$$

To evaluate the BS over all time points, the Integrated Brier Score (IBS) is used, which averages the BS over a range of times up to $t_{\max}$:

(8) $$\mathrm{IBS}\left(t_{\max}\right)=\frac{1}{t_{\max}}\int_{0}^{t_{\max}}\mathrm{BS}\left(t\right)\;dt.$$

This integral form ensures that the evaluation of the BS is not restricted to a specific time point, providing a more comprehensive measure of the prediction accuracy. A lower IBS value indicates better model performance, reflecting more accurate predictions of survival probabilities across the considered time range. The pec package (version 2023.04.12) in R was used to implement these calculations. The average number of features selected by each model was also calculated, along with the frequency at which the algorithm failed to converge and select features for predictor construction.

Kaplan–Meier estimation is a non-parametric method that estimates the survival function from time-to-event data, and is a widely used method in survival analysis to visualise the proportion of individuals surviving over time [30]. The log-rank test is often used in conjunction to compare survival curves between two or more groups [31]. Kaplan–Meier survival analysis was conducted, with samples categorised into high- and low-risk based on the median prognostic index (PI), as defined in Equation (2). To ensure the median was an appropriate threshold for this categorisation, the distribution of PI values was inspected and found to be unimodal, which supported the suitability of the median as a cutoff. Survival differences between these groups were assessed using the log-rank test.

### 2.6. External Validation with the CGGA Dataset

To assess the generalisability of our findings beyond the TCGA dataset, an external validation was conducted using data from the CGGA. Specifically, the mRNAseq_693 dataset was used. The CGGA cohort was classified according to the WHO-2016 guidelines, where, as noted by Mendonça et al. [2], key histological features such as microvascular proliferation or necrosis were required for the diagnosis of GBM. Thus, updating the classification to align with the WHO-2021 criteria only required excluding GBM samples without an *IDH* wild-type status. The mRNAseq_693 dataset included 693 patients. For this study, the following inclusion criteria were applied: (1) patients diagnosed with GBM or recurrent GBM (rGBM), (2) patients who had mRNA-seq data, (3) patients who had survival data, and (4) the availability of an *IDH* status. A total of 227 patients met these criteria, of which 182 exhibited an *IDH* wild-type status and were therefore considered GBM cases under the WHO-2021 classification. Gene expression values were normalised using the same preprocessing steps as described previously.

### 2.7. Enrichment Analysis

The R package clusterProfiler (version 4.10.1) [32] was used to identify Gene Ontology (GO) terms and Kyoto Encyclopedia of Genes and Genomes (KEGG) pathways for the selected features. Additionally, the ReactomePA (version 1.46.0) [33] package was employed to perform a pathway enrichment analysis based on the Reactome database. This analysis aimed to provide insights into the biological processes and pathways associated with the most relevant genes identified in our study.

## 3. Results

All analyses and computations in this study were performed using the R software environment (version 4.3.0; R Core Team, 2023). The code developed for this analysis is open source and available at https://github.com/sysbiomed/GBMsurvival (accessed on 7 April 2025).

### 3.1. Feature Selection and Model Performance

To assess the variability in feature selection and performance, the entire pipeline was repeated 10 times using different random splits while consistently maintaining a 70% training/30% testing ratio and preserving the proportion of censored data in each set. This iterative approach provided a more reliable estimation of the model stability and generalisability.

After the data processing, the initial number of features was 19,465. Applying univariate Cox regression to pre-select survival-associated features reduced this number to 2345 ± 813.9 (considering the 10 replicates). Next, the RSF and Boruta algorithms were applied. For the RSF-based approach, an RSF model was trained to compute the Variable Importance (VIMP) scores. These scores were analysed to understand their distribution and to inform the selection of a meaningful threshold value for filtering out non-informative features before training the multivariate regularised Cox models.

**Figure 1:**
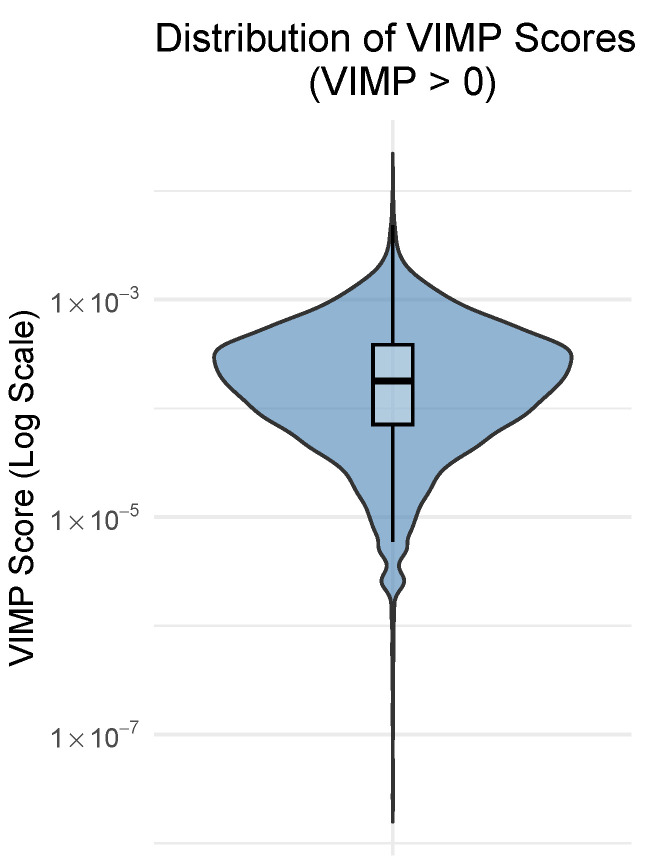
Distribution of Variable Importance (VIMP) scores computed by the Random Survival Forest (RSF) model across 10 seeds. Only positive VIMP values are included.

To guide the threshold selection, we examined the distribution of VIMP scores across the 10 replicates. Half of the features (51%) had VIMP scores less than or equal to zero. VIMP values that are near zero or negative correspond to noisy variables, whereas larger positive values reflect a meaningful predictive contribution. To gain clearer insight into this distribution, we visualised the data using a violin plot on a logarithmic scale (Figure 1) for only the VIMP scores greater than zero. When plotted on a linear scale, the distribution appeared extremely compressed, making it difficult to distinguish informative features. In contrast, the log-scale plot more clearly revealed the heavy right tail of the distribution, where a small proportion of features exhibited substantially higher importance. These visualisations reinforced the necessity of applying a threshold to isolate a compact, high-contribution subset of features for downstream modelling.

**Figure 2:**
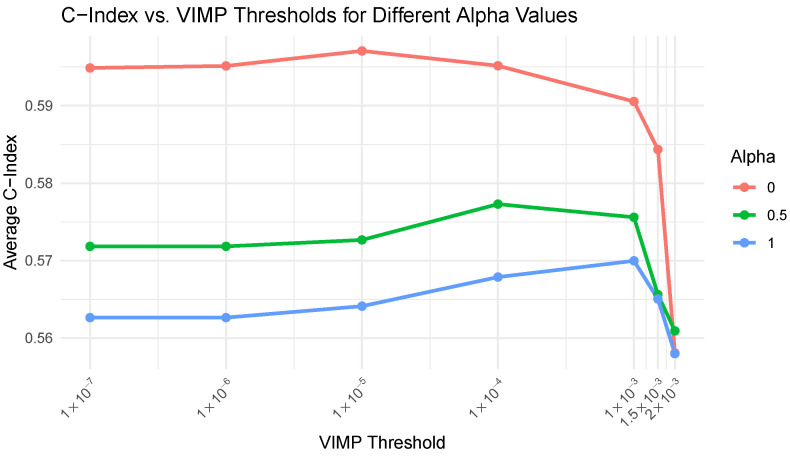
Average concordance index (C-index) for different Variable Importance (VIMP) score threshold values applied to the Cox regularised models trained with the Random Survival Forest (RSF)-selected features. This VIMP score threshold value determined the minimum importance a feature needed to be selected for training the Cox model. The results were averaged over 10 replicates with α values of 0 (Ridge), 0.5 (Elastic Net) and 1 (LASSO).

We then systematically optimised the feature VIMP threshold, for which we selected the features to train the regularised Cox model. For each threshold value, the C-index was computed and averaged across the 10 replicates, with regularisation strengths defined by α values (0 for Ridge, 0.5 for Elastic Net, and 1 for LASSO). The results, summarised in Figure 2, show that although the C-index slightly decreased when the VIMP threshold increased from 0.0001 to 0.001, the performance loss was modest compared with the substantial gain in the model sparsity. A threshold of 0.0001 resulted in 758.4 ± 169.3 selected features, whereas 0.001 reduced this number to just 59.3 ± 12.7. On average, this threshold retained only 2.5% of the features previously pre-selected by univariate Cox regression, which reduced the number from approximately 2345 ± 813.9 to a compact yet informative subset. This simplification improved the model interpretability while preserving the strong predictive performance across all regularisation settings. Therefore, the 0.001 threshold was selected as the most appropriate cut-off point for downstream model training.

After optimising the feature selection threshold using the regularised Cox model trained with the RSF-selected features, we further explored the Boruta algorithm for feature selection. The Boruta algorithm was applied to the training set, and two scenarios were evaluated: one where only the model’s confirmed features, i.e., those identified as significantly important compared with the shadow features, were used to train the Cox model and another where both the confirmed and tentative features were included. The tentative features were those for which the algorithm could not conclusively determine importance, often due to fluctuating importance scores or convergence limits, leaving them without a definitive classification. For each case, the models were optimised across the same regularisation strengths, and the performance was evaluated by averaging the C-index across 10 replicates. The results showed that regardless of the regulariser applied, incorporating both the confirmed and tentative features led to a higher C-index compared with using only the confirmed features. This may have been because some tentative features still carried relevant prognostic information that contributed to the model performance, even if their individual importance was not conclusively determined by the Boruta algorithm.

For the models that applied HubCox and OrphanCox regularisation, neither univariate Cox regression nor any additional feature selection techniques were applied.

After training, the models were evaluated using the C-index, IBS and the average number of coefficients retained in the models. The percentage of non-convergence cases was also recorded. For a robust performance assessment, all the models were evaluated across 10 different replicates. The results are summarised in Table 1.

Among the models that employed LASSO regularisation, the LASSO–Cox model trained with the Boruta-selected features achieved the highest C-index (0.578 ± 0.055) and a good IBS (0.129 ± 0.017) while maintaining a small number of coefficients (7.4 ± 2.9) and no non-convergence cases. Similarly, for the Elastic Net regularisation, the Elastic Net–Cox model trained with the Boruta-selected features demonstrated a comparable performance, where it achieved a C-index of 0.578 ± 0.053 and an IBS of 0.129 ± 0.016. For the Ridge regularisation, the Ridge–Cox model achieved the highest overall C-index (0.595 ± 0.052) and the lowest IBS (0.127 ± 0.015) but retained a much larger number of coefficients (2345 ± 813.9), as Ridge does not perform feature selection and instead shrinks all coefficients without eliminating any.

### 3.2. Risk Stratification and Biological Relevance

To evaluate the ability of the models to stratify patients into risk groups, a Kaplan–Meier survival analysis was performed. Survival curves were generated for high-risk and low-risk groups using the models trained on the entire dataset. For RSF, we applied the previously identified VIMP score threshold of 0.001, while for Boruta, both the confirmed and tentative features were included.

**Figure 3:**
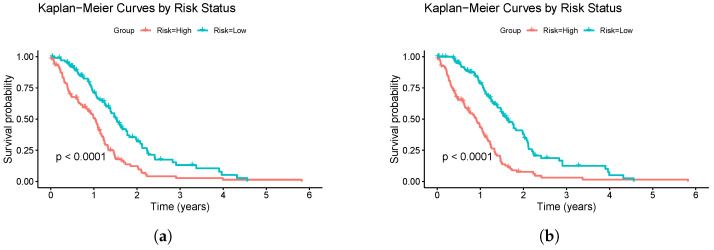
Kaplan–Meier survival curves for high-risk and low-risk groups based on models trained on the entire dataset. The survival outcomes predicted by the (**a**) Elastic Net–Cox trained with the Boruta-selected features and (**b**) Ridge–Cox trained with the RSF-selected features. The risk groups were defined by the median risk score, with the curves reflecting the survival trends across the entire dataset. The statistical significance of the group differences was evaluated using the log-rank test.

The Cox models that did not incorporate feature selection via RSF or Boruta were excluded from this analysis because when the number of features was small, it exhibited worse performance than the other models and often failed to converge. When they did achieve better performance, it retained an excessively large number of features, making it impractical for further analysis. For each remaining model type, the regulariser that achieved the best performance (based on the average C-index) was selected. The risk groups were divided based on the median risk score predicted by each model. The results are shown in Figure 3.

**Figure 4:**
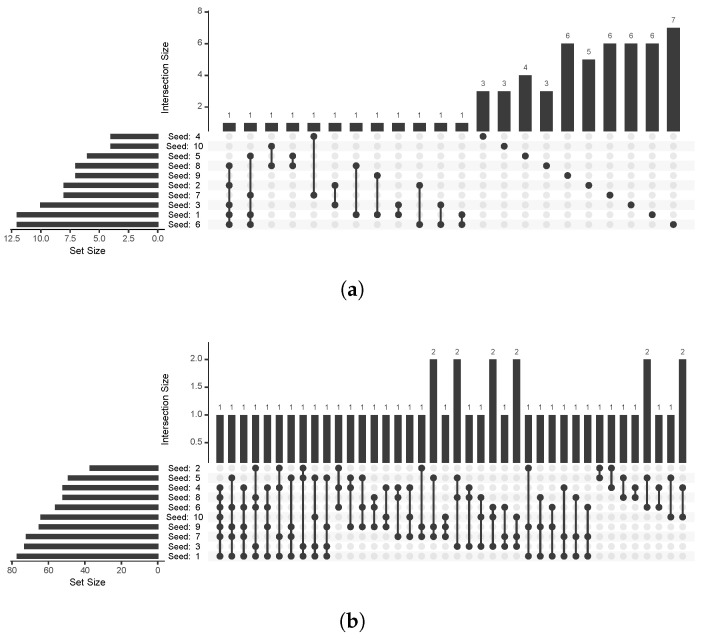
Upset plots illustrating the consistency of feature selection across the 10 replicates. (**a**) Elastic Net–Cox trained with the Boruta-selected features and (**b**) Ridge–Cox trained with the RSF-selected features. The rows represent individual replicates, and the vertical bars indicate the number of features consistently selected across different combinations of replicates. A limit of 40 intersection bars was applied to maintain interpretability, fully covering the Elastic Net–Cox trained with the Boruta-selected features but only partially representing the Ridge–Cox trained with the RSF-selected features due to the larger number of selected features.

To evaluate the consistency of gene selection across different replicates, we examined the overlap of features selected by the models. Figure 4 displays the results, where the rows correspond to the 10 replicates, denoted by seeds, and the vertical bars at the top represent the intersection sizes. These bars indicate how many genes were consistently selected across various combinations of replicates, with a larger bar reflecting a higher overlap. To ensure the plot remained interpretable, a limit of 40 intersection bars was applied. This threshold was sufficient for the Elastic Net–Cox trained with the Boruta-selected features but not for the Ridge–Cox trained with the RSF-selected features due to the substantially larger number of selected features. Consequently, the figure is not exhaustive for the Ridge–Cox trained with the RSF-selected features, underrepresenting its full intersection dynamics.

To quantify the overlap across replicates, we computed the Jaccard index for each of the 10 replicates by dividing the number of genes shared between both models (i.e., the intersection) by the total number of unique genes selected by either model (i.e., the union). The resulting Jaccard index averaged 0.0896 ± 0.0282, indicating limited but consistent overlap between the two selection strategies. Additionally, to assess whether the observed overlap in the gene selection between the two methods was greater than that expected by chance, Fisher’s exact test was performed independently for each replicate. For each replicate, a 2×2 contingency table was constructed using the gene pool available to both methods. The table summarised the number of genes selected by both methods, genes selected only by Elastic Net–Cox with Boruta features, genes selected only by Ridge–Cox with RSF features and those not selected by either. Fisher’s exact test was then used to determine whether the observed overlap was significantly higher than expected under the null hypothesis of independence between the two selection methods. The resulting *p*-values were consistently low across the replicates, with an average of $2.94\times 10^{-6}$ and a standard deviation of $3.84\times 10^{-6}$, indicating a statistically significant overlap in the gene selection between the two approaches.

To further analyse the consistency of gene selection between the Boruta and RSF-based approaches, the genes identified by Elastic Net–Cox trained with the Boruta-selected features and Ridge–Cox trained with the RSF-selected features across 10 replicates were compared. Table 2 lists the genes selected by both methods in at least two different replicates. The counts indicate the number of replicates in which each gene was selected by each method, highlighting the overlap and consistency in the feature selection between the two approaches.

In addition, the gene Ribosomal Protein S27a (*RPS27A*) consistently emerged as the sole predictor of patient risk across nearly all predictors using the HubCox PPI, regardless of whether LASSO or Elastic Net regularisation was applied.

### 3.3. External Validation and Functional Enrichment

**Figure 5:**
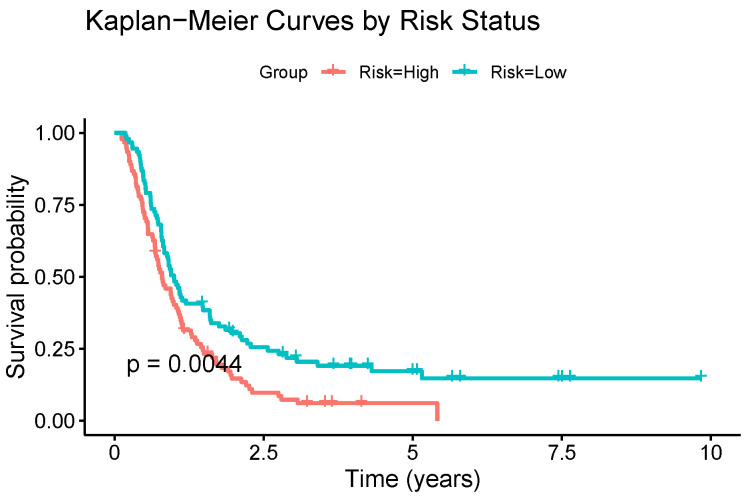
Kaplan–Meier survival curves of high-risk and low-risk groups based on the model trained with the CGGA GBM dataset updated according to the WHO-2021 classification using the selected genes. The risk groups were defined by the median risk score, with the curves reflecting survival trends across the entire dataset. The statistical significance of the group differences was evaluated using the log-rank test.

To assess the generalisability of the selected genes beyond the TCGA dataset, we conducted an external validation using the Chinese Glioma Genome Atlas (CGGA). The expressions of the nine genes listed in Table 2 were extracted and used to fit a multivariate Cox regression model. The PI (Equation (2)) derived from this model was then used to stratify patients into high- and low-risk groups based on the median PI value. The Kaplan–Meier survival curves revealed a statistically significant difference in the overall survival between these two groups (*p*-value = 0.0044, log-rank test), as shown in Figure 5.

KEGG, GO and Reactome pathway enrichment analyses were performed separately on the genes listed in Table 2, on the genes selected by the Elastic Net–Cox models trained with the Boruta-selected features and the Ridge–Cox models trained with the RSF-selected features. For these models, the analyses considered the genes identified when training with the entire dataset, as well as the combined set of genes selected across all the replicates. Only the Reactome pathway enrichment analyses performed on the genes selected by the Elastic Net–Cox model trained with the Boruta-selected genes using the entire dataset yielded significant results. Of the six genes in the analysis, only four (TIMP Metallopeptidase Inhibitor 1—*TIMP1*, Insulin Like Growth Factor Binding Protein 6—*IGFBP6*, Lysyl Oxidase Like 1—*LOXL1* and Listerin E3 Ubiquitin Protein Ligase 1—*LTN1*) were annotated in the Reactome database; C1q And TNF Related 9B—*C1QTNF9B* and Ankyrin Repeat Domain 33—*ANKRD33* were not associated with any known Reactome pathway. The enriched pathways were the Regulation of Insulin-like Growth Factor (IGF) transport and uptake by Insulin-like Growth Factor Binding Proteins (IGFBPs) (gene *TIMP1* and *IGFBP6)*; adjusted *p*-value = 0.0129) and the extracellular matrix organisation (gene *TIMP1* and *LOXL1*; adjusted *p*-value = 0.0364).

## 4. Discussion

This study aimed to evaluate the usefulness of employing Boruta and RSF algorithms combined with traditional regularised Cox regression, as well as the application of the network-based regularisation methods HubCox and OrphanCox to develop a prognostic signature for distinguishing high-risk and low-risk GBM patients. The analysis focused on assessing the methods’ effectiveness in generating interpretable and robust models.

Cox regression with Ridge regularisation achieved the highest average C-index, 0.595 ± 0.052. Nonetheless, the model’s retention of a significantly large number of features, 2345 ± 813.9, rendered it less feasible for clinical or biological interpretation.

In contrast, the Boruta and RSF feature selection methods significantly reduced the number of features while maintaining a competitive average C-index. Boruta combined with Elastic Net achieved a strong balance between the model performance and interpretability, where it achieved an average C-index of 0.578 ± 0.053 with only 7.8 ± 2.9 features retained. Similarly, RSF paired with Ridge–Cox demonstrated a robust performance, where it achieved a higher C-index of 0.588 ± 0.051 while selecting 59.6 ± 12.7 features. Notably, models that incorporated Boruta and RSF also demonstrated comparable IBS values, highlighting their ability to maintain the prediction accuracy.

The network-based regularisation methods HubCox and OrphanCox demonstrated a superior prediction accuracy, as evidenced by their best-in-class IBS values (0.123 and 0.125, respectively) while utilising a reduced number of features. However, the lower C-index scores for both HubCox and OrphanCox revealed limitations in their ability to accurately rank patients by risk. Additionally, like traditional Cox models, these methods encountered convergence issues in certain replicates. Notably, when employing HubCox, the gene *RPS27A* emerged as the sole risk predictor in all the models with LASSO regularisation and nearly all models (7 out of 8) with Elastic Net regularisation, highlighting its distinct prominence. Recognised as a proto-oncogene, *RPS27A* is involved in critical cellular processes, including cell cycle regulation, apoptosis and proliferation. Its dysregulation has been linked to multiple cancer types [34]. In gliomas, elevated *RPS27A* expression has been associated with more aggressive gliomas [35]. While the biological relevance of *RPS27A* is well-supported, its dominance across nearly all network-based regularised models raises concerns about whether these methods genuinely exploit the underlying biological network structure or are instead biased towards highly connected hub genes, potentially at the expense of identifying a more functionally diverse set of prognostic markers.

These results highlight the value of combining advanced feature selection techniques with regularised Cox regression to create interpretable and high-performing prognostic models. Among the approaches evaluated, models that integrated RSF and Boruta with regularised Cox regression demonstrated the optimal balance between performance and feature reduction while consistently achieving convergence. Further analyses focused exclusively on these models.

The Kaplan–Meier survival analysis provided additional insights into the ability of the models to stratify patients into high- and low-risk groups (Figure 3). Both the Elastic Net–Cox model trained with the Boruta-selected features and the Ridge–Cox model trained with the RSF-selected features effectively stratified the patients, presenting significant separation between the high- and low-risk groups (*p*-value < 0.0001). However, as time progressed, the separation between the survival curves diminished. This was likely due to the declining number of patients under observation over time, which reduced the statistical power to detect the differences between groups. This limitation underscores the challenges of long-term survival analysis in datasets with significant right-censoring. After analysing the survival curves, the median survival times were estimated to be approximately 1.0 years for the high-risk groups and around 1.6 years for the low-risk groups.

When evaluating the consistency of feature selection, as shown in Figure 4, the Elastic Net–Cox trained with the Boruta-selected features appears to select a smaller, more frequently recurring set of genes across multiple replicates. In contrast, the Ridge–Cox trained with the RSF-selected features exhibited greater variability in the selected genes. However, this apparent consistency of Boruta may have been partly attributed to the smaller number of features it selected, which naturally reduced the variability across replicates.

To assess the significance of gene overlap between the Boruta and RSF approaches, we applied Fisher’s exact test across the replicates, confirming that the observed intersection was statistically significant and unlikely to occur by chance. The most frequently co-selected genes across replicates are listed in Table 2, highlighting candidates with potential biological relevance. Notably, genes such as *LOXL1* and *IGFBP6* stood out for their high selection frequencies, underscoring their potential significance as robust prognostic markers. *LOXL1* and *IGFBP6* have already been associated with glioma survival in the literature [36,37]. Yu et al. [36] reported that *LOXL1* functions as an important mediator that increases the antiapoptotic capacity of tumour cells, suggesting that targeting *LOXL1* could be a potential strategy for treating glioma. Similarly, Bei et al. [37] demonstrated that *IGFBP6* acts as an inhibitor of tumour cell survival and migration, with its expression inversely correlating with the glioma grade. Beyond these two genes, TNF Receptor Superfamily Member 18 (*TNFRSF18*) and Matrix Metallopeptidase 19 (*MMP19*), also selected by both methods, have been associated with a poor prognosis in glioma [38,39]. The identification of these genes by both methods suggests a convergence on biologically meaningful features. This overlap reinforces the reliability of these genes as potential targets for further investigation and clinical application and also illustrates the advantages of our proposed methodology to identify interesting prognostic biomarkers for glioblastoma.

To further investigate the functional roles of the identified genes, KEGG, GO and Reactome pathway enrichment analyses were conducted on the gene sets derived from the Elastic Net–Cox model trained with the Boruta-selected genes, Ridge–Cox model trained with the RSF-selected genes and the set of genes listed in Table 2. Among all the analyses, only the Reactome enrichment based on the Boruta–Elastic Net–Cox model trained on the full dataset yielded statistically significant results. Two pathways were significantly enriched: Regulation of Insulin-like Growth Factor (IGF) transport and uptake by Insulin-like Growth Factor Binding Proteins (IGFBPs) and extracellular matrix organisation. These findings highlight potential roles for IGF signalling and extracellular matrix remodelling in the biological processes captured by the model, although the small number of contributing genes warrants a cautious interpretation.

To evaluate the generalisability of the genes listed in Table 2 beyond the TCGA dataset, we conducted an external validation using the Chinese Glioma Genome Atlas (CGGA). A multivariate Cox regression model was fitted using the expression of the nine genes listed, and the resulting prognostic index (PI) was used to stratify the patients into high- and low-risk groups based on the median PI. The Kaplan–Meier survival analysis revealed a statistically significant separation between the two groups (*p*-value = 0.0044), as shown in Figure 5. These results demonstrate the robustness and potential transferability of the identified gene signature, suggesting its applicability across independent GBM cohorts and reinforcing its relevance for future clinical investigations.

## 5. Conclusions

This study comprehensively evaluated survival prediction models for glioblastoma (GBM) by integrating statistical techniques, traditional regularisation, network-based regularisation and machine learning algorithms.

The results demonstrate that while traditional Ridge–Cox regression achieved the highest predictive performance in terms of the C-index, indicating a superior patient risk ranking, its reliance on an excessively large number of features limits its practical utility for clinical and biological applications. Conversely, the network-based regularisation methods HubCox and OrphanCox allowed for the selection of a reduced number of features and achieved superior IBS values, indicating their higher prediction accuracy, even if their C-index performance was lower.

Among the evaluated models, those that integrated the RSF and Boruta emerged as the most effective in balancing the performance and interpretability by retaining a small number of features while maintaining competitive predictive performance. The Kaplan–Meier survival analysis demonstrated the efficacy of these models in stratifying GBM patients into distinct high- and low-risk groups. However, the gradual convergence of the survival curves over time highlights the inherent limitations of long-term survival analysis, particularly in datasets with substantial right-censoring.

An analysis of feature selection consistency revealed that Boruta and RSF models identified several common features across replicates. Notably, genes such as *LOXL1*, *IGFBP6*, *TNFRSF18* and *MMP19*, identified by both methods, have established links to glioma survival. The consistent selection of these genes underscores their potential as prognostic markers and valuable targets for future therapeutic exploration.

These common genes selected across both methods were used to fit a multivariate Cox model and construct a prognostic index (PI) on an external validation dataset from the CGGA. The resulting PI successfully stratified CGGA patients into high- and low-risk groups, with the Kaplan–Meier analysis revealing a significant survival difference (*p*-value = 0.0044), supporting the robustness and transferability of the signature to an independent GBM cohort.

In conclusion, this study underscored the potential of integrating statistical and machine learning approaches with classical survival analysis methods to enhance prognostic modelling. The results highlight the need to balance the predictive performance and interpretability, paving the way for further refinements in survival analysis methodologies.

## Figures and Tables

**Table 1:** Models’ performance summary. Average and standard deviations of the concordance index (C-Index), Integrated Brier Score (IBS) and number of coefficients (Nb of Coeffs). Non-Conv stands for non-convergence.

Model	C-Index	IBS	Nb of Coeffs	% Non-Conv
LASSO (α=1)				
Cox	0.548 (0.057)	0.130 (0.015)	10.2 (9.1)	30
RSF + Cox	0.569 (0.057)	0.131 (0.016)	12.2 (4.4)	0
Boruta + Cox	0.578 (0.055)	0.129 (0.017)	7.4 (2.9)	0
HubCox PPI	0.557 (0.061)	0.123 (0.012)	1 (0)	20
OrphanCox PPI	0.535 (0.049)	0.125 (0.011)	6 (3.1)	20
Elastic Net (α=0.5)				
Cox	0.565 (0.054)	0.128 (0.015)	17.9 (12.6)	20
RSF + Cox	0.574 (0.049)	0.131 (0.015)	16.3 (5.4)	0
Boruta + Cox	0.578 (0.053)	0.129 (0.016)	7.8 (2.9)	0
HubCox PPI	0.557 (0.061)	0.123 (0.012)	1.4 (1.1)	20
OrphanCox PPI	0.551 (0.048)	0.125 (0.010)	8.3 (4.1)	20
Ridge (α=0)				
Cox	0.595 (0.052)	0.127 (0.015)	2345 (813.9)	0
RSF + Cox	0.588 (0.051)	0.130 (0.016)	59.6 (12.7)	0
Boruta + Cox	0.577 (0.051)	0.128 (0.016)	10.1 (4.2)	0

**Table 2:** Comparison of genes selected by Elastic Net–Cox trained with the Boruta-selected features and Ridge–Cox trained with the RSF-selected features across 10 replicates. The genes listed were selected by both methods in at least two different replicates.

Gene Name	Abbreviation	Boruta Count	RSF Count
Lysyl Oxidase Like 1	*LOXL1*	5	5
Insulin Like Growth Factor Binding Protein 6	*IGFBP6*	4	6
Protocadherin Beta 3	*PCDHB3*	2	3
Ro60, Y RNA Binding Protein	*RO60*	2	3
Cell Adhesion Molecule 3	*CADM3*	2	3
TNF Receptor Superfamily Member 18	*TNFRSF18*	2	3
Centrosomal Protein 97	*CEP97*	2	7
*Nitric Oxide Synthase Trafficking*	*NOSTRIN*	2	4
*Matrix Metallopeptidase 19*	*MMP19*	2	2

## Data Availability

The R code developed for this analysis is open source and available at https://github.com/sysbiomed/GBMsurvival (accessed on 7 April 2025). The datasets analysed during the current study are publicly available from The Cancer Genome Atlas (TCGA) database (https://www.cancer.gov/tcga) (accessed on 12 December 2023) and Chinese Glioma Genome Atlas (CGGA) database (accessed on 10 April 2025)). The disease classification using the WHO-2021 taxonomy guidelines is available at https://github.com/sysbiomed/MONET (accessed on 16 April 2024)).
